# Emotions behind a mask: the value of disgust

**DOI:** 10.1038/s41537-023-00388-3

**Published:** 2023-09-14

**Authors:** Marina A. Pavlova, Jonas Moosavi, Claus-Christian Carbon, Andreas J. Fallgatter, Alexander N. Sokolov

**Affiliations:** 1 Department of Psychiatry and Psychotherapy, Tübingen Center for Mental Health (TüCMH), Medical School and University Hospital, Eberhard Karls University of Tübingen, Tübingen, Germany; 2 Department of General Psychology and Methodology, University of Bamberg, Bamberg, Germany

**Keywords:** Human behaviour, Schizophrenia

## Abstract

The impact of face masks on social cognition and interaction became a popular topic due to the long-lasting COVID-19 pandemic. This theme persists in the focus of attention beyond the pandemic, since face covering not only reduces the overall amount of face information available but also introduces biases and prejudices affecting social perception at large. Many questions are still open. One of them is whether gender of beholders affects inferring of emotions covered by face masks. Reading covered faces may be particularly challenging for individuals with mental disorders, most of which are gender-specific. Previous findings are not only sparse, but inconclusive because most research had been conducted online with resulting samples heavily dominated by females. Here in a face-to-face study, females and males were presented with a randomized set of faces covered by masks. In a two-alternative forced-choice paradigm, participants had to indicate facial emotions displayed by posers. In general, the outcome dovetails with earlier findings that face masks affect emotion recognition in a dissimilar way: Inferring some emotions suffers more severely than others, with the most pronounced influence of mask wearing on disgust and close to ceiling recognition of fear and neutral expressions. Contrary to our expectations, however, males were on overall more proficient in emotion recognition. In particular, males substantially excelled in inferring disgust. The findings help to understand gender differences in recognition of disgust, the forgotten emotion of psychiatry, that is of substantial value for a wide range of mental disorders including schizophrenia. Watch Prof. Marina Pavlova discussing this her work and this article: https://vimeo.com/860126397/5966610f49?share=copy.

## Introduction

The impact of face masks on social cognition and interaction became a valuable and popular research topic due to the long-lasting COVID-19 pandemic with compulsory face-masks-wearing safety regulations. This theme remains in the focus of research attention beyond the pandemic, since face coverings not only reduce the overall amount of face information but introduce perceptual biases and prejudices affecting efficient social interaction and mental health at large^1,2^.

For inferring most emotional expressions (in particular, subtle), complementary information flows from the upper and lower face parts are desirable. Indeed, in daily life, beholders habitually have access to a plenty of facial cues. Yet, it is assumed the lower part of a face is essential for the recognition of happiness and disgust, the upper portion for anger and fear, and both for surprise and sadness^3,4^. Already initial studies in the field indicated that not all emotions are equally affected by medical face masks covering the lower portion of a face^2,5^. Irrespective of differences in cultural/ethnical background (East Asians prioritize global information and fixate more on the center of a face, the nose area, and less on the eyes and mouth areas than Westerners^6–8^), digital superimposing masks on photographs of faces persistently leads to a substantial decrease in inferring sadness, and, in particular, disgust as well as their perceived intensity and confidence in recognition^9–27^. On the same wavelength, in UK residents of different ethnicity (Caucasian, Black, and Asian/Pacific observers), face masks are reported to primarily hamper inferring disgust and sadness, also having substantial impact on the recognition of happiness^28^. In Turkish university students, neither effects of mask pattern (angular or curvy) nor color (black or white) on facial emotion recognition was found, with the most pronounced influence of all types of masks on afraid/fearful and disgusted faces^13^. The findings obtained with the separate groups tested in May 2020 and July 2021 indicate that the unfavorable influence of face masks on sadness and disgust recognition still persists after more than a year of the pandemic^14^, or, in other words, the impact of habituation or experience with masked faces on face reading appears to be rather negligible. Noteworthy, comparable effects of face covering are obtained using female faces expressing emotions with a face mask and in faces with a mask artificially imposed onto face photographs (except anger), with the poorest recognition of disgust and sadness^16^. Brief exposure (for 250 s) to masked faces results in a basically similar pattern of results, with recognition of facial disgust affected most strongly, along with a rather limited impact on recognition of anger^29^.

**Fig. 1:**
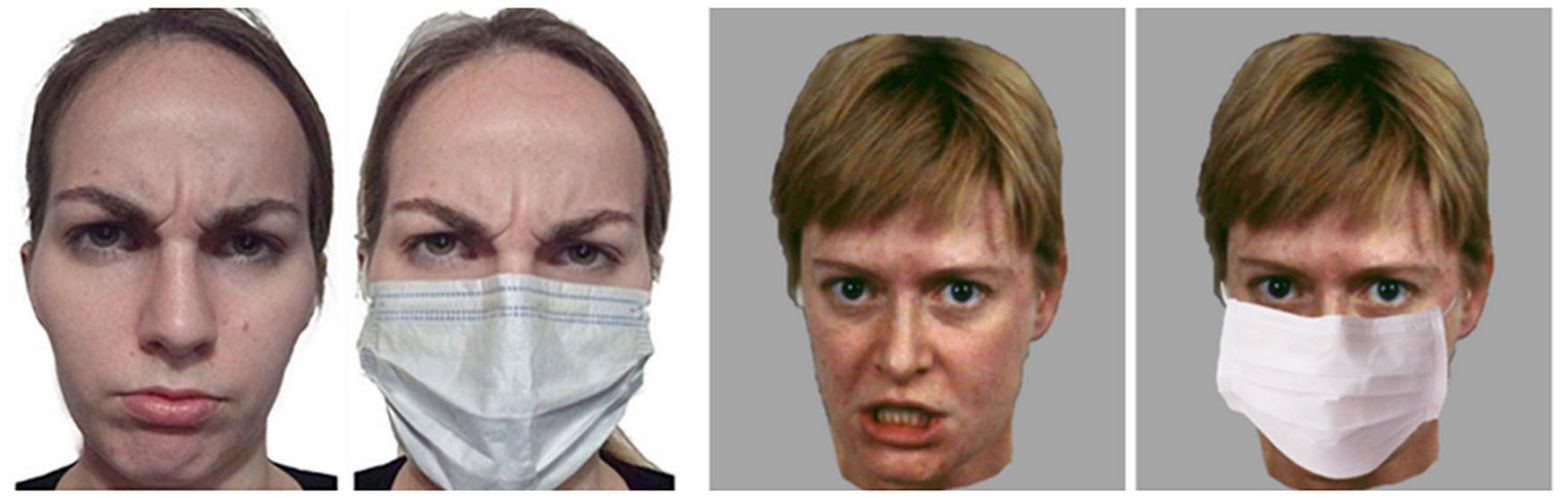
Differently expressed anger by posers with diverse cultural background. Anger expressed with different involvement of the upper and lower face parts in an Italian poser (left panel; from Proverbio and Cerri, 2022, Front Neurosci; the Creative Commons Attribution [CC BY] license) and in a face taken from the Vienna Emotion Recognition Task, VERT-K, with subsequent modification (right panel; from Grahlow et al., 2022, PLoS ONE; the Creative Commons Attribution [CC BY] license) may lead to different conclusions by studying facial expressions under unusual viewing conditions, for example, when hidden behind a mask.

There is much less harmony concerning emotional expressions most resistant to face masks wearing. Experimental studies underscore neutral expressions^9,11,14,21,24,28^ and happiness^19^. The primacy of anger in the sense of its robustness against face masks is also emphasized^20,24,26,30^, albeit the opposite effects are described as well^9,10,12,15,17,29^. This discrepancy may be attributable to methodological issues, in particular, differences in emotion expression by posers (Fig. 1) or faces databases used such as the MPI FACES database or Radboud Faces Database. In some studies, visual input for emotional impressions is hardly comparable in terms of head tilts, rolls, and yaws. Moreover, cultural differences in emotion expression and experience may contribute to inconsistency of the findings. For example, face masks hamper recognition of happiness in US American but not in Japanese individuals^31^.

The pattern of results similar to the effects obtained with static photographs is demonstrated using more ecologically valid faces in motion. In videos of dynamic faces, masks impair inferring sadness, disgust, and happiness, leaving neutral expressions, fear, and social (fake/dishonest/polite) smiling largely untouched^32,33^. Face masks affect ratings of the extent to which reward, affiliation, and dominance smiles in moving faces convey positive feelings, reassurance, and superiority, respectively^34^. Fairly unexpectedly, however, social smiles are reported to appear more honest in masked than unmasked dynamic faces^33^. Under usual viewing conditions, social smiles are determined primarily by information from the mouth with indifferent *cold* eyes, whereas shining warm eyes make *real* smiles^35^. Yet, even covered by masks true smiles are rated as happy and pleasant or, in other words, the glow of real smiles still shows^36^. However, hidden behind masks happiness is often mistaken for neutral expressions, a poker face^37^. Noteworthy, individuals with higher empathic concern demonstrate higher recognition levels for disgust in masked faces, but not for other basic emotions^29^. Principally, this agrees with the findings that emotional intelligence as well as self-reported emotional intelligence do not affect emotion recognition in both masked and unmasked faces^14^. In the same vein, both affective and cognitive empathy are not tied with emotion recognition in masked faces^27^.

Reading covered faces may be particularly challenging for individuals with mental, neurological, and psychosomatic disorders characterized by deficient non-verbal social cognition already in the pre-pandemic period^2^. However, the data is extremely sparse and controversial. Most neuropsychiatric conditions are gender- (a social construct reflecting social norms, roles, biases, and practices) and/or sex- (a neurobiological construct) specific, possessing a skewed ratio: females and males are differently affected in terms of prevalence, clinical manifestation, and symptom severity. Major depressive disorder (MDD) shows a female preponderance with around twice as many women affected as men^38^. By contrast, in schizophrenia (SZ), males are more often affected with a ratio ranging from 1.4 to 1.6 with an earlier age of onset, worse premorbid functioning, and a greater severity of negative symptoms^39^. Moreover, males and females with SZ may possess distinct profiles in social cognition and metacognition^40,41^.

The question arises whether individuals with mental disorders exhibit gender differences in reading faces covered by masks? To date, only a handful of studies address this issue even in typically developing (TD) individuals, and the outcome is inconclusive. The primary reason is that most studies have been conducted online with samples heavily predominated by females. This makes revealing gender differences questionable: gender comparisons in unbalanced samples may lead to paradoxical statistical outcomes. Studies with designs balanced in respect to gender either report the absence of gender differences in static^14,17,42^ and dynamic masked faces^34^, or reveal female superiority in reading covered faces^42,43^. Females rate negative emotions covered by face masks as more negative, and positive emotions as more positive than males^44^.

Covering faces with masks leaves a comparable amount of visual information for face reading as the Reading the Mind in the Eyes Test (RMET) that contains a set of photographs of a pair of the eyes along with the surrounding part of a face^2,45^. Most recent work indicates that the RMET predicts the accuracy of facial affect recognition in masked faces, whereas the Tromsø Social Intelligence Scale (TSIS) does not^46^. Considering well-documented (small, but reliable) female proficiency in reading language of the eyes as assessed by the RMET^2,45,47^, one can expect that females are also more skillful in reading emotions in masked faces. The present work intended to clarify whether gender of perceivers affects inferring emotions in faces covered by face masks.

## Methods

### Participants

Overall, 53 participants (25 females and 28 males) were engaged in the study. The data sets of two male participants had to be discarded, since routine check prior to data analysis revealed that they were outliers with the overall recognition accuracy beyond ±3 standard deviations (SDs) of other male participants. Thus, the data of 26 male participants entered the data processing. None of them had head injuries, a history of mental disorders (including autism spectrum disorders (ASD), SZ, and MDD), or regular drug intake (medication). Males were aged 23.69 ± 3.90 years (mean ± SD; median, Mdn, 23 years, 95% confidence interval, CI [23.08, 24.31]; age range 18–33 years), and females 22.28 ± 3.34 years (Mdn, 22 years, 95% CI [21.74, 22.82]; age range 18–31 years), with no age difference between the groups (Mann-Whitney test, *U* = 261, *p* = 0.230, two-tailed, n.s.). As performance on the task required a proficient language command, German as native language (mother tongue) served as an inclusion criterion. We also strived for homogeneity in respect to cultural background that can potentially affect reading of masked faces^31^. The number of participants was determined prior to the study by demands of statistical data processing. As in previous work^48–51^, gender was self-identified by participants; there were also no female participants with extreme masculine appearance and behavior, and vice versa. All observers had normal or corrected-to-normal vision. Participants were run individually and were naïve as to the purpose of the study. None had previous experience with such displays and tasks. The study was conducted in line with the Declaration of Helsinki and approved by the local Ethics Committee at the University of Tübingen Medical School. Informed written consent was obtained from all participants. Participation was voluntary, and the data sets were processed anonymously.

### Face stimuli, task, and procedure

**Fig. 2:**
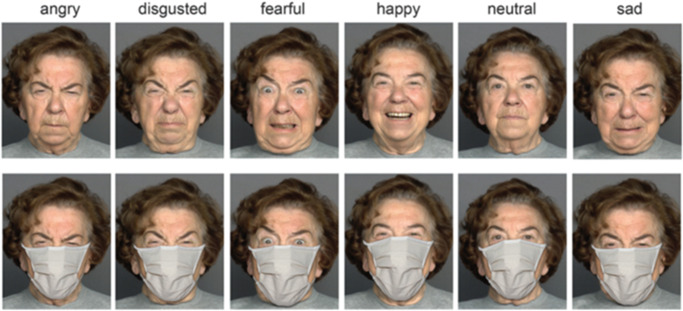
A female poser expressing six basic emotions. Faces are shown under full-face (top) and covered-by-mask conditions (bottom row). From Carbon, Front. Psychol. (2020), the Creative Commons Attribution [CC BY] license. These images are presented for illustrative purposes only, and had not been used as experimental material in the present study.

The original face stimuli without masks were taken from the MPI FACES database^52^ with the project-specific permission, and then modified by superimposing face masks with a graphics editor^9^. Frontal photographs of six (three female and three male) Caucasians were used from three distinct age groups (young, middle, and older age). Each depicted person displayed six emotional states (anger, disgust, fear, happiness, sadness, and neutrality; Fig. 2). A typical face mask in beige (a so-called “community mask” commonly used during the COVID-19 pandemic) was applied by means of a graphics editor to all faces and adapted individually to properly fit the specific face. Realistic shadow effects were added to improve the naturalistic impression of the images with masked faces (Fig. 2). The stimulus set comprised 36 images (6 emotions × 2 genders × 3 age groups) repeated three times per session, resulting in a total of 108 trials. As the task was designed for later use in patients, unlike^9,14^, we used only two (one correct and one incorrect) rather than all possible six alternative responses for emotion recognition. Using only two response alternatives leads to a considerable decrease in task difficulty (in the sense of decision-making complexity as well as reliance on language proficiency and comprehension) and test duration, both of which are welcome in examination of patients. The response alternative pairs were chosen based primarily on the emotion confusion data^9,14^: angry— disgusted, fearful—sad, and neutral—happy. For avoiding possible transfer and passive leaning effects on emotion recognition, we used masked faces only.

**Fig. 3:**
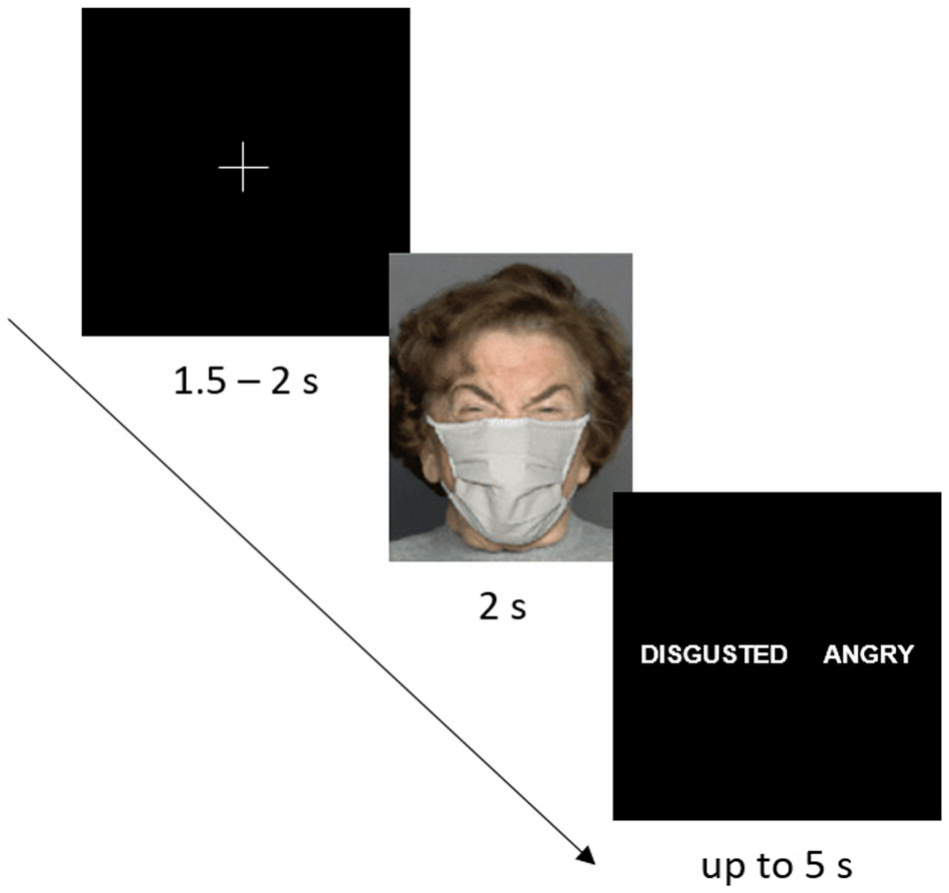
Schematic representation of experimental procedure. Each trial started with presentation of a white fixation cross in the middle of the screen for 1.5–2 s followed by 2-s presentation of one out of six facial emotional expressions hidden behind a mask. After stimulus presentation, participants had to indicate, within 5 s in a 2AFC task, the displayed emotion by choosing one response option among two alternatives (correct and incorrect response; for example, either disgusted [correct] or angry [incorrect] facial expression). The face image is presented for illustrative purposes only, and had not been used as experimental material in the present study.

Participants were administered a computer version of the emotion recognition task by using Presentation software (Neurobehavioral Systems, Inc., Albany, CA, USA). The stimuli subtended a visual angle of 9.8° × 9.8° at an observation distance of 70 cm. They were presented in a pseudo-randomized order, one at a time for 2 s in three runs separated by short breaks. A schematic representation of experimental procedure (each trial) is given in Fig. 3. Upon image offset, two words (correct and incorrect responses) appeared on the right and left sides of a black screen. The correct response position varied randomly across trials. Participants were asked to respond as accurately but also as fast as possible once a response screen was on (with a time limit of 5 s). On each trial, they had to indicate a displayed emotion by pressing a respective key on the side of correct response. Once a response was given (or else the time limit elapsed), a white fixation cross appeared for a duration jittered between 1.5 and 2 s prior to the start of next trial. Instructions were carefully explained to participants and their understanding had been proven with pre-testing (about ten trials) performed under supervision of an examiner. No immediate feedback was provided to participants. The testing lasted for about 15 min.

### Data processing and analysis

Prior to statistical data processing, normality of data distributions was routinely examined by using Shapiro-Wilk tests with subsequent use of either parametric (for normally distributed data sets) or non-parametric statistics. For not normally distributed data sets, additionally to means and SDs, Mdns and 95% CIs are reported. Statistical inference was accomplished by means of mixed-model analyses of variance (ANOVAs, the outcome of which is reported to be resistant to normality of data distribution^53–55^) and post-hoc pairwise comparisons by using Tukey’s honestly significant difference (HSD) tests with software package JMP (Version 16, SAS Institute, Cary, NC, USA). Non-parametric statistics (Mann–Whitney test and Wilcoxon signed-rank test) were performed for between- and within-group comparisons, respectively, with MATLAB (version 2022a; MathWorks Inc., Natick, MA, USA).

## Results

### Recognition accuracy

**Fig. 4:**
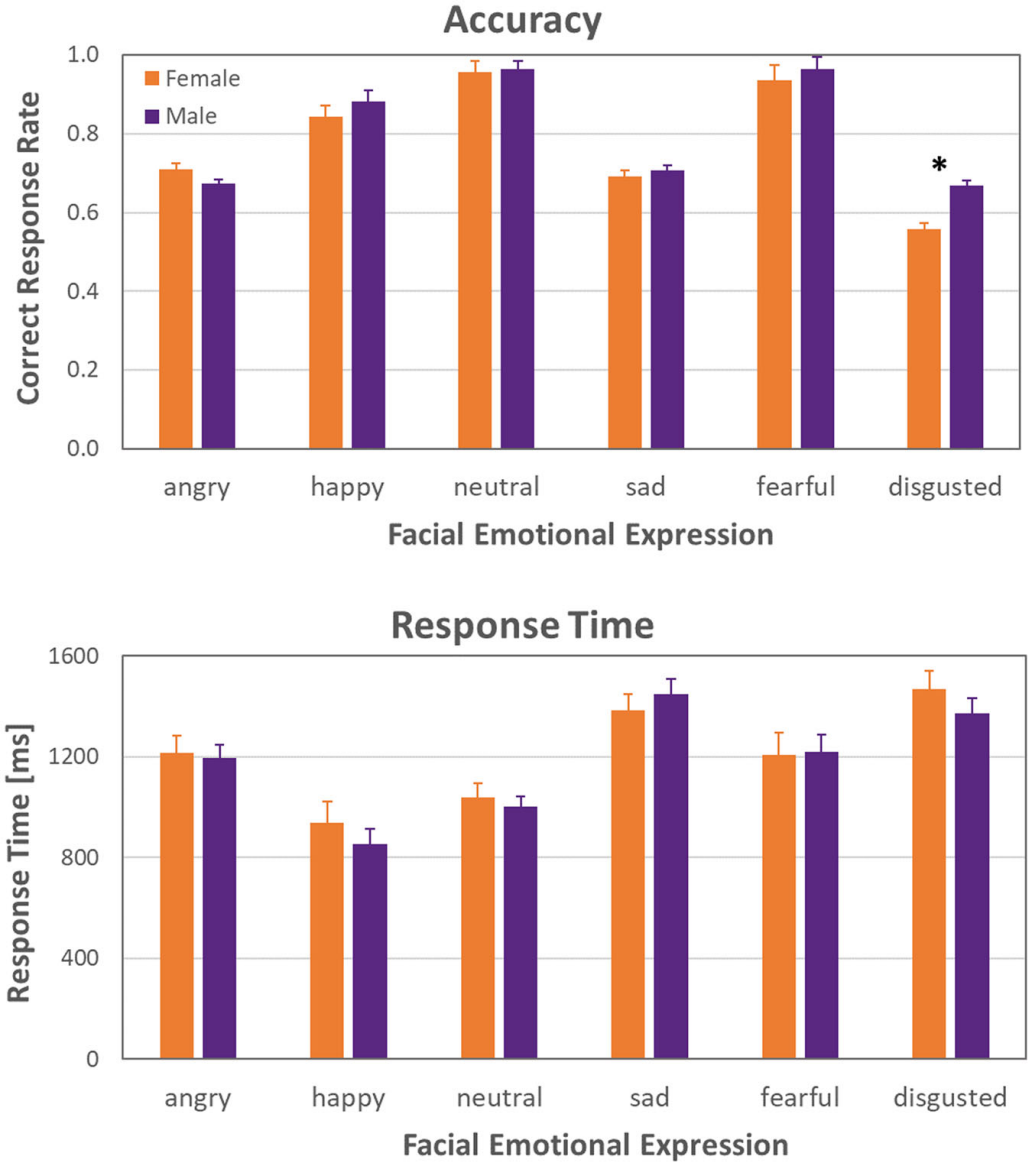
Gender impact on reading emotions in masked faces. Mean correct response rate (top panel) and mean response time, RT (bottom panel) for recognition of facial emotions hidden behind a mask in female (orange) and male (violet) participants. Vertical bars represent ±SEM. Asterisks indicate significant differences (*p* < 0.05).

Individual correct response rates were submitted to a two-way mixed-model ANOVA with the within-subject factor Emotional Expression (angry, fearful, neutral, disgusted, happy, and sad) and between-subject factor Observer Gender (female/male). A main effect of Gender was significant (*F*(1,245) = 4.17, *p* = 0.042; effect size, eta-squared *η*^2^ = 0.079), albeit, contrary to our expectations, with a higher emotion recognition accuracy in males than in females (Fig. 4). A main effect of Emotional Expression was highly significant (*F*(5,245) = 78.15, *p* < 0.0001; effect size, *η*^2^ = 0.615). A Gender by Emotion interaction tended to reach significance (*F*(5,245) = 2.14, *p* = 0.062).

In accord with previous findings (see “Introduction” section), the outcome shows that face masks disproportionally affect facial emotional recognition: the most recognizable (close to the ceiling level of performance, Fig. 4) were neutral expression, fear, and happiness, whereas disgust, sadness, and angriness turned out to be least recognizable. As this analysis is beyond the focus of the present study, the outcome of post-hoc pair-wise comparisons (two-tailed Tukey HSD, multiplicity adjusted) is provided in Supplementary Material (Tables S1–S2). As seen from Table 1 summarizing the outcome of the least squares mean analysis (expressions with no differences in recognition are marked by the same letter), the most recognizable were neutral expressions and fear (without any difference between them, both marked by A), followed by happiness (marked by B), and then by sadness and anger (without any difference between them, both marked by C). Disgust was the most poorly recognizable emotion.

**Table 1:** Least squares mean rates for recognition of emotions behind a mask.

Emotion		Least squares mean
Neutral	A	0.960
Fearful	A	0.949
Happy	B	0.862
Sad	C	0.700
Angry	C	0.691
Disgusted	D	0.613

Note: Top to bottom, the most to least recognizable emotions; emotions with no significant differences in recognition are marked by the same letter.

**Fig. 5:**
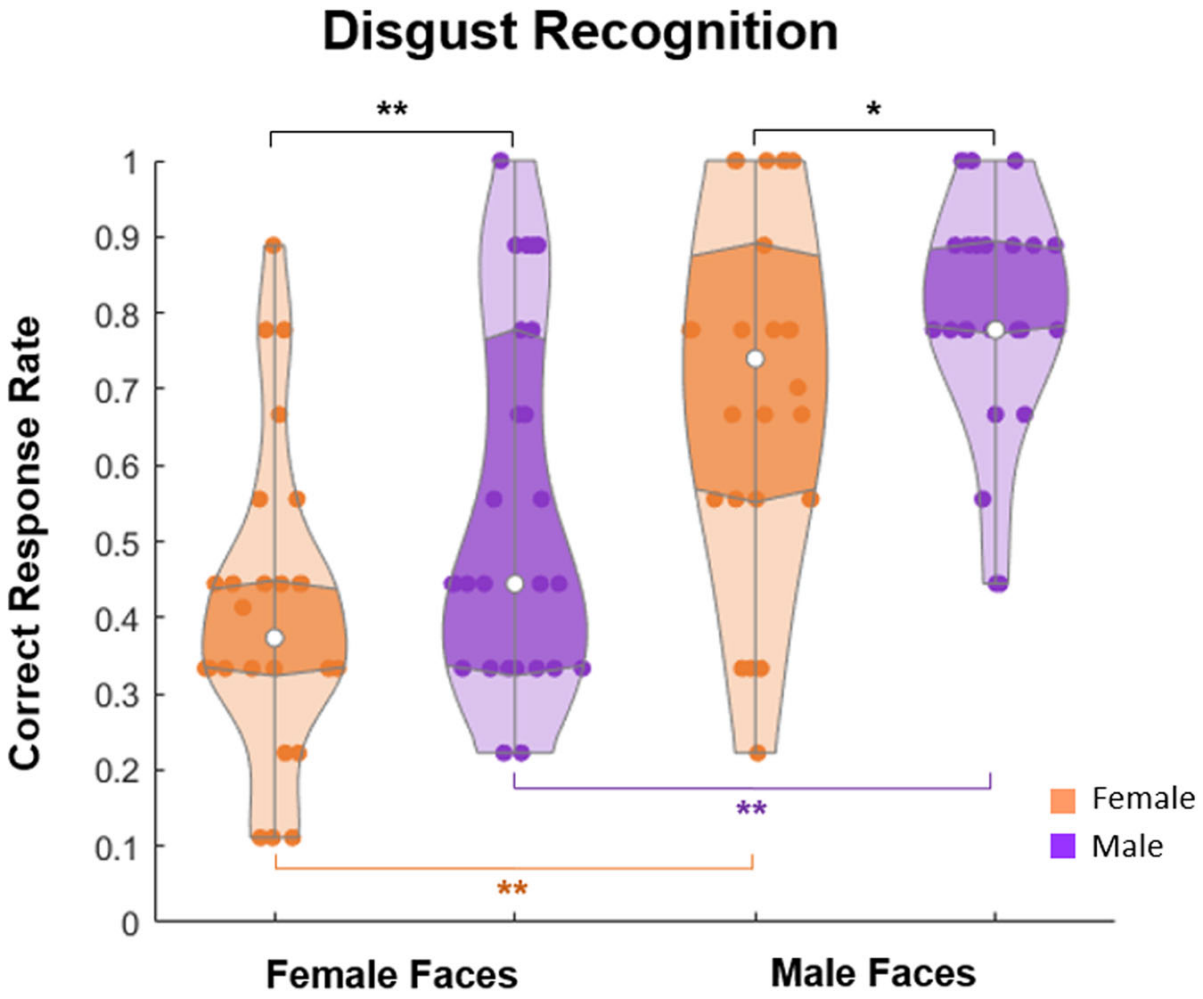
Violin plot of disgust recognition rates in masked female and male faces. Mean correct response rate for recognition of disgust hidden behind a mask in female (orange) and male (violet) participants separately for female and male posers. Vertical bars represent ±SEM. Double asterisks indicate significant differences (*p* < 0.05), single asterisk indicates a tendency (*p* = 0.07).

Contrary to our expectations, males were more proficient than females in recognition of disgust (males, 0.61 ± 0.12, and females, 0.56 ± 0.19; *t*(49) = 3.34, *p* = 0.044, corrected, *p* = 0.007, uncorrected; Tukey HSD corrected, two-tailed; effect size Cohen’s *d* = 0.954). As seen in Fig. 4, no gender differences occurred for all other emotions. A further analysis (performed separately for female and male faces) showed that mostly female faces behind a mask contributed to hitches in disgust recognition in both female and male beholders (Fig. 5). Both female and male participants recognized disgust in female masked faces substantially poorer than in male faces (for females, Wilcoxon signed-rank test, *z* = 3.59, *p* < 0.0003, two-tailed, effect size *d* = 2.063; for males, *z* = 3.50, *p* < 0.0005; two-tailed, effect size *d* = 1.888). As expected, males excelled on recognition of disgust in female faces and tended to be more proficient in male faces (for female faces, Mann–Whitney test, *U* = 231, *p* = 0.039; for male faces, *U* = 247, *p* = 0.072).

### Response time

Individual response time (RT) values for correct responses were submitted to a two-way mixed-model ANOVA with the within-subject factor Emotional Expression (angry, fearful, neutral, disgusted, happy, and sad) and between-subject factor Observer Gender (female/male). Note, the analysis of RT plays only a secondary role, since participants had been asked to respond as soon as possible after the stimulus offset. A main effect of Gender was not significant (*F*(1,245) = 1.22, *p* = 0.270, n.s.), whereas a main effect of Emotional Expression was highly significant (*F*(5,245) = 57.33, *p* < 0.0001; effect size, *η*^2^ = 0.539). As seen in Fig. 4, in accord with the recognition accuracy analysis, the fastest responses were given for neutral expression, fear, and happiness, while RTs for disgust and sadness were longer. A Gender by Emotion interaction failed to reach significance (*F*(5,245) = 0.97, *p* = 0.436, n.s.). Pair-wise comparisons did not reveal any gender differences in RT for all emotions.

## Discussion

This work was aimed at investigation of gender impact on the recognition of facial emotions hidden behind a mask. The outcome indicates that: (i) Masks hamper recognition (both accuracy and RT) of emotions in a different way: while some emotions such as happiness, fear, and neutral expressions remain rather well recognizable even when a face is hidden behind a mask, others such as anger, sadness and, in particular, disgust are poorly recognizable. This is in close agreement with previous research^2,5^. (ii) Contrary to our expectations, however, males were, in general, more proficient in facial emotion recognition, in particular, in recognition of disgust behind a mask, than females. A large body of earlier studies analyzing gender impact on reading covered faces has been conducted online with samples heavily predominated by females. Comparison of gender differences in unbalanced samples may result in paradoxical statistical outcomes. A few studies (with designs balanced in respect to gender) either report the absence of gender differences^14,17^, or reveal female superiority in reading covered faces^43^. In particular, women are reported to show a better performance for subtle expressions such as surprise and sadness, both in masked and whole-face conditions, and men excel in recognition of fear, especially in masked faces^20^. Research on reading language of the eyes as assessed by the RMET also implies female superiority in reading masked faces^45^. However, it was not the case in the present study. Noteworthy, males are reported to be more proficient than females in recognition of emojis (especially negative ones), while females are better in recognition of natural facial expressions^56^. The lack of fine-grained structure in emojis appears to be more favorable for males. This strategy may be also more profitable for males while reading covered by masks faces. (iii) Furthermore, compared to masked male faces, disgust represented in female faces is particularly poorly recognizable by both female and male beholders. These items will be discussed further in turn.

### Why is disgust in masked faces recognized so poorly?

It is widely believed that the eyes represent the window to the soul^45^. Yet, in the same vein as previous research, the present findings indicate that (i) not all emotions are equally affected by face masks covering the lower portion of a face, and (ii) digital superimposing masks on photographs of faces consistently results in a substantial decrease in inferring sadness and disgust as well as their perceived intensity and confidence in recognition^9–27^. The most probable reason for this is that disgust is expressed primarily by the lower part of a face, namely, by a mouth and a nose^3,4^. In particular, disgust expressions are predominately comprised of the nose wrinkle, lip corner pullers, and lower lip depressor^34,57,58^. In accord with this, longer fixation on the mouth positively ties with recognition accuracy of disgust (as well as anger)^59^. Furthermore, disgust recognition benefits more than other basic emotions from audiovisual information as compared with video-only or audio-only conditions^60^. An area of the eyes and surrounding regions may be rather comparable when expressing sadness, disgust, and anger, sharing similar activation of muscles of the upper face part (Fig. 2). This may lead to perceptual errors of mistaking these emotions for one another^9,14,21,23^.

### Why do males excel in disgust recognition? Why is disgust less recognizable in female faces?

Recognition of disgust is heavily affected by a face mask in both female and male observers, albeit it is easily recognizable by healthy people in unmasked faces^9,14^. The present study shows that disgust recognition is affected in women more severely than in men. One may ask why males are more proficient in reading disgust in covered faces. One possible explanation would be that females and males use different gender-dependent perceptual strategies. For example, eye tracking indicates that females look at the eyes before looking at the mouth for angry, happy, and surprised, but not for disgusted, fearful or sad facial expressions^61^. When information from a mouth is absent (hidden behind a mask), this strategy may be inefficient for disgust recognition. Different perceptual styles (either global/holistic or local) may also account for gender differences in reading disgust in masked faces. It is believed that females possess a rather holistic perceptual style, whereas males a rather local one with an effortful piecemeal analysis of facial features and cues^51,62–67^. Obviously, face covering more heavily affects the holistic style requiring appearance of faces in their entirety, whereas the local information processing style allows extracting some subtle cues pointing to disgust in the upper part of faces. This is in line with the study in Japanese individuals revealing that people who are capable of inferring complex mental states of others from *subtle* cues may be less susceptible to the negative impact of mask wearing^25^. Moreover, individuals with a higher empathic concern demonstrate higher recognition level for disgust in masked faces^29^. Reportedly, women not only experience emotional disgust more often, but also spend more time attending to disgust facial expressions than men^68^. In our opinion, however, aversive behavior toward disgust seems to be more plausible. In line with this, as compared to female SZ patients, male individuals with SZ excel in recognition of disgust in unmasked faces^69^. Apparently, this agrees with the present findings indicating that reading disgust in masked female faces may be more demanding than in male faces, and for female as compared to male observers. One possible account for this may be that female posers express disgust even to a greater degree by the lower part of a face than male posers as well as even less by the upper part of a face. These assumptions, however, call for further experimental support.

### Reading covered faces in mental and neurological disorders

As mentioned earlier, reading covered faces may be particularly challenging for individuals with mental, neurological, neurodevelopmental, and psychosomatic disorders^2^. However, experimental evidence is sparse. Among patients with MDD, SZ, bipolar disorder (BD), and TD individuals, patients with MDD and SZ exhibit most difficulties in identifying subtle (but not intense) expressions of happiness^70^. While masks heavily impact recognition of happiness and sadness in TD and ASD persons, reading of anger is unaffected in both groups. Yet, disgust recognition in covered by masks faces is similarly diminished in both groups^71^. In the absence of high levels of comorbid alexithymia (difficulties in identifying and describing emotions experienced by oneself or others), no evidence is reported for deficient emotion recognition with masked faces in ASD^72^. By contrast, healthy individuals with higher scores on the AQ-10 (the 10-item Autism Spectrum Quotient) are less accurate and confident in facial expression recognition, perceiving emotional expressions as less intense^12^. Yet, another study in TD individuals reveals that reading emotions in masked faces is unrelated to alexithymia as assessed by the 20-item Toronto Alexithymia Scale as well as to autistic traits expression as measured by the Autism Spectrum Quotient^23^. Individuals with developmental prosopagnosia (DP, a neurodevelopmental condition characterized by lifelong deficits in face recognition of neural and genetic origins^73^) exhibit the same level of facial emotion recognition of unmasked faces as neurotypical controls, but demonstrate deficits in subtle emotion recognition in masked faces, in particular, mistaking happiness for neutral expression^74^. Facial emotion recognition is affected by masks in cognitively unimpaired relapsing-remitting patients with multiple sclerosis (MS); these patients also exhibit selective impairments in recognition of fear both in unmasked and masked faces^75^.

In a nutshell, the primary novel outcome of the present study indicates that males are not over-performed by female peers in reading basic emotions in covered by masks faces. In particular, reading disgust in masked faces is more demanding for females than for males, and for female than for male faces expressing disgust. These findings may help to explain gender/sex differences in disgust recognition that is of substantial value for understanding and treatment of mental disorders. Disgust is considered *the forgotten emotion of psychiatry*^76^ that *explains everything*^77^. Moreover, disgust is one of the primal emotions that define a uniquely human social cognitive domain^57,58^. Most important, a wide range of mental disorders (anxiety disorders, obsessive-compulsive disorder (OCD), specific phobias, depression, eating disorders, and body dysmorphia) are characterized by alterations in expression and/or recognition of disgust. In response to images (non-face, scenic) eliciting disgust, individuals with SZ exhibit alterations in functional magnetic resonance imaging (fMRI) brain activation, in particular, hyperactivation of the right temporal cortex^78^. In response to briefly exposed facial disgust expressions, reduced fMRI activation of the insula is reported in patients with SZ; moreover, this activation is positively linked to social loneliness and negatively tied with agreeableness^79^. For understanding the value of disgust for mental health, many exciting research avenues remain to be explored.

### Supplementary information

Tables S1-S2

## Data Availability

The data supporting the conclusions of this paper are either included in the paper or will be made available by the authors upon request to any qualified researcher.
