# Supplementary material for: Emotions behind a mask: the value of disgust
Source: Schizophrenia (Heidelb). 2023 Sep 14;9(1):58. doi: 10.1038/s41537-023-00388-3 (PMC10502067; doi:10.1038/s41537-023-00388-3)
Supplement: Supplementary file 1 — Tables S1-S2 [file 41537_2023_388_MOESM1_ESM.docx]

|  |  |  |  |  |  |
| --- | --- | --- | --- | --- | --- |
|  | **Emotion Pair** | | **t value** | **P** |  |
|  | Anger | Happy | - 7,25 | < 0.001 |  |
|  | Anger | Neutral | - 11,44 | < 0.001 |  |
|  | Anger | Sad | - 0,38 | 0.999 |  |
|  | Anger | Fear | - 10,96 | < 0.001 |  |
|  | Anger | Disgust | 3,29 | 0.015 |  |
|  | Happy | Neutral | - 4,19 | < 0.001 |  |
|  | Happy | Sad | 6,87 | < 0.001 |  |
|  | Happy | Fear | - 3,71 | 0.004 |  |
|  | Happy | Disgust | 10,54 | < 0.001 |  |
|  | Neutral | Sad | 11,07 | < 0.001 |  |
|  | Neutral | Fear | 0,48 | 0.997 |  |
|  | Neutral | Disgust | 14,73 | < 0.001 |  |
|  | Sad | Fear | - 10,58 | < 0.001 |  |
|  | Sad | Disgust | 3,67 | 0.004 |  |
|  | Fear | Disgust | 14,25 | < 0.001 |  |
|  |  |  |  |  |  |

|  | **Neutral** | **Fear** | **Happy** | **Sad** | **Anger** | **Disgust** |
| --- | --- | --- | --- | --- | --- | --- |
| **Neutral** |  | 0.997 | < 0.001 | < 0.001 | < 0.001 | < 0.001 |
| **Fear** | 0.997 |  | 0.004 | < 0.001 | < 0.001 | < 0.001 |
| **Happy** | < 0.001 | 0.004 |  | < 0.001 | < 0.001 | < 0.001 |
| **Sad** | < 0.001 | < 0.001 | < 0.001 |  | 0.999 | 0.004 |
| **Anger** | < 0.001 | < 0.001 | < 0.001 | 0.999 |  | 0.015 |
| **Disgust** | < 0.001 | < 0.001 | < 0.001 | 0.004 | 0.015 |  |

Significant values are given in green.

**Table S2.** Consolidated matrix of statistical differences (two-tailed HSD p-values, multiplicity adjusted) between emotion expressions as provided in Table S1

**Table S1.** Pairwise comparisons (Tukey HSD, two-tailed, multiplicity adjusted) for an ANOVA main effect of Emotional expression recognition.
